# Thrombolysis in a Child with Acute Arterial Ischemic Stroke without Large Vessel Occlusion

**DOI:** 10.1017/cjn.2020.1

**Published:** 2020-03

**Authors:** Ilana Hanes, Serena Orr, Jorge Davila, Adam Kirton, Erick Sell

**Affiliations:** Faculty of Medicine, University of Ottawa, Ottawa, Canada; Section of Neurology, Department of Pediatrics, Alberta Children’s Hospital, The University of Calgary, Calgary, Canada; Department of Medical Imaging, Children’s Hospital of Eastern Ontario, Ottawa, Canada; Departments of Pediatrics and Clinical Neurosciences, Cumming School of Medicine, University of Calgary, Calgary, Canada; Division of Neurology, Department of Pediatrics, Children’s Hospital of Eastern Ontario, University of Ottawa, Ottawa, Canada

A 14-year-old male experienced thunderclap headache, dysarthria, and expressive aphasia followed by complete loss of vision and consciousness. He arrived to hospital 25 min after onset. Initial assessment found no verbal response, bilateral withdrawal to painful stimuli, and rightward gaze. A non-contrast computed tomography scan performed 40 min after arrival was normal. PedNIHSS was 23 given decreased level of consciousness, inability to answer questions or follow commands, sustained rightward gaze, and no effort in movement against gravity (Table 1).

**Table 1:** NIHSS before and after administration of IV tPA

NIHSS item	Before IV tPA	After IV tPA
(1a) LOC	3	0
(1b) LOC questions	2	0
(1c) LOC commands	2	0
(2) Best gaze	1	0
(3) Visual	UN	0
(4) Facial palsy	0	0
(5a) Motor left arm	3	0
(5b) Motor right arm	3	0
(6a) Motor left leg	3	0
(6b) Motor right leg	3	0
(7) Limb ataxia	UN	2
(8) Sensory	2	0
(9) Best language	3	1
(10) Dysarthria	UN	2
(11) Extinction and inattention	0	0
Total	25	5

Magnetic resonance imaging (MRI) obtained 70 min after arrival revealed areas of diffusion restriction within the right cerebellar hemisphere, the posterior aspect of the cerebellar vermis and both thalami. Time-of-flight MR angiogram (MRA) showed patency of all major cerebral arteries (Figure 1).

**Figure 1:**
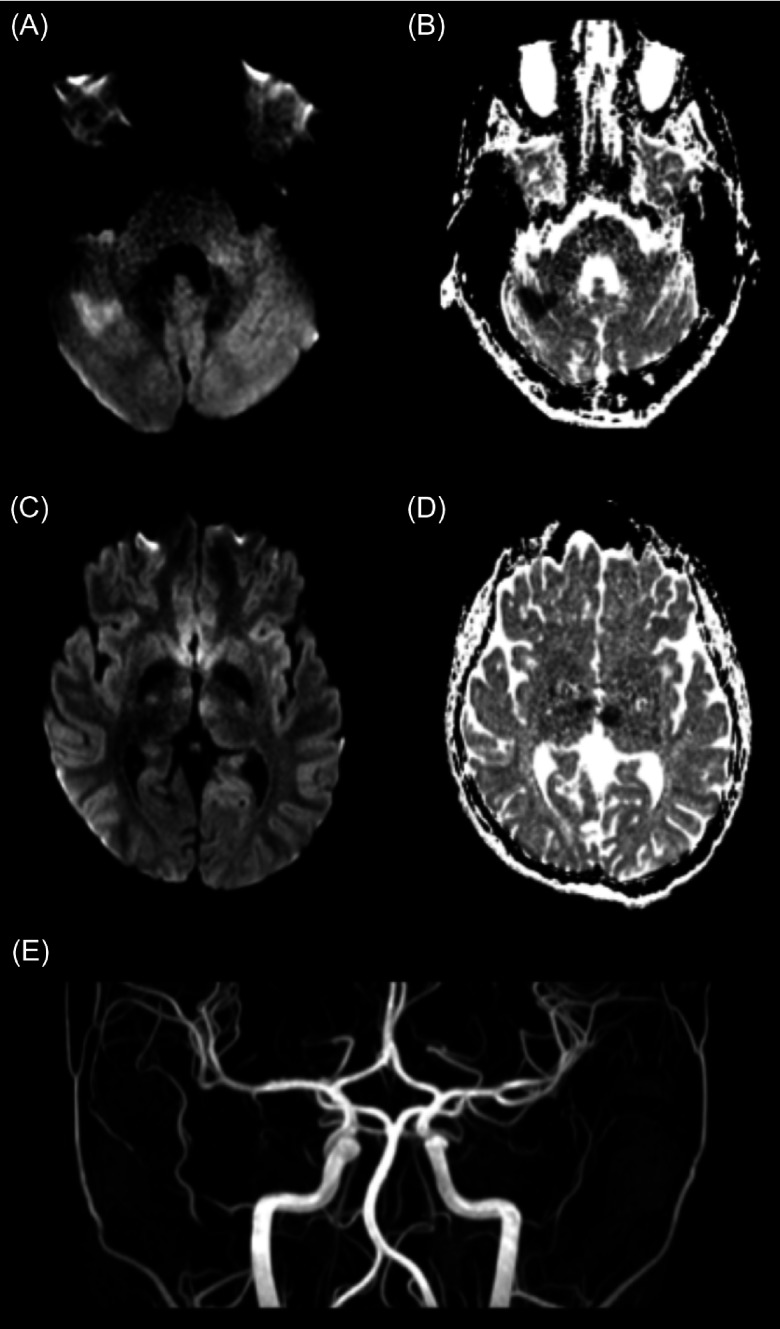
Diffusion-weighted imaging (DWI) and ADC images acquired 1 h and 10 min after presenting to hospital, approximately 1 hr and 35 min after onset of symptoms, demonstrating areas of schema in the right cerebellar hemisphere and both the right and left thalami. (A) DWI at the level of the cerebellum demonstrating diffusion restriction in the right cerebellar hemisphere. (B) Corresponding ADC demonstrating lower ADC values in corresponding areas to the diffusion restriction on DWI. (C) DWI at the level of the thalami demonstrating diffusion restriction in the right and left thalamus. (D) Corresponding ADC demonstrating lower ADC values in the corresponding areas to the diffusion restriction DWI. (E) MRA demonstrating patency of all arteries.

The patient was treated with intravenous tissue plasminogen activator (IV tPA) despite patency of all large vessels. Treatment was started 3 h 20 min after arrival to hospital. The patient received IV tPA 0.9 mg/kg total, with 10% of the total dose given over the first 5 min and the remainder infused over 1 h.

He regained consciousness. PedNIHSS following thrombolysis was 5 given bilateral limb ataxia, moderate aphasia, and severe dysarthria (Table 1). No deficits were discernible on exam 48 h later.

Repeat MRI showed expected evolution of the areas of ischemia and no interval bleeding (Figure 1). The patient was started on unfractionated heparin and transitioned to enoxaparin. He was discharged home 6 d later, immediately returning to all activities of daily living.

Investigations performed for coagulopathy, rheumatological, infectious or metabolic causes of stroke, electrocardiogram, echocardiogram, and bilateral leg ultrasound for deep vein thrombosis were unremarkable except for transthoracic echocardiogram revealing a small patent foramen ovale shunting left to right.

Stroke is a rare neurological disease in children. While prognosis is generally superior to that following adult stroke, morbidity following pediatric arterial ischemic stroke (AIS) is substantial.^1^ Diagnosis of pediatric stroke is challenging and options for acute intervention are limited. Therapies that have revolutionized adult stroke remain undefined in children.

Many retrospective reviews have evaluated the rate of use of thrombolysis in pediatric AIS. One particular study suggested a rate of 3% thrombolysis in pediatric AIS.^2^ This study also highlighted the shortcomings of evidence for thrombolysis in pediatric stroke including frequent administration outside recommended time intervals, substantial rate of poor outcomes, including death or neurological deficit, and risk of associated intracranial hemorrhage.^2^ A larger retrospective study found a similar rate of thrombolysis in pediatric AIS.^3^ The overall mortality was 4.7%, but there were no fatal cases among those treated with IV tPA. Approximately 5% of patients treated with IV tPA had secondary intracranial hemorrhage; however, none were clinically significant.^3^ This suggested that greater rates of poor outcome, again defined by death or neurological deficit, were related to pretreatment morbidity rather than complications of thrombolysis.^3^ Such evidence fails to provide clear direction for the use of thrombolysis in pediatric AIS.

Thrombolysis is not approved by the US Food and Drug Administration or Health Canada for pediatric AIS. Current published stroke guidelines from the American Heart Association and American College of Chest Physicians comment that thrombolysis is not indicated in the pediatric population and should not be used outside clinical trials.^4,5^ The Australian Clinical Consensus guideline for diagnosis and acute management of pediatric stroke states that thrombolysis “may be appropriate in specific children,” and recommends consensus-based eligibility criteria [i.e., those from the “Thrombolysis in Pediatric Stroke Study” (TIPS) trial] and dosing (i.e., based on adult dosing guidelines).^6^ The TIPS trial was a multisite, randomized controlled, dose-escalation trial intended to determine safety, dosing, and feasibility of IV tPA in pediatric AIS.^7^ Initially funded by the NIH/NINDS in 2010, the TIPS trial closed by December 2013 due to the lack of recruitment.^7^ Though the TIPS trial inclusion and exclusion criteria are used to guide clinical practice, they have not been validated for this purpose.^7^ The feasibility of treatment of pediatric stroke is demonstrated by the successful use of a stroke protocol in the pediatric population.^8^ Following this protocol, no intracranial or peripheral bleeding with recanalization strategies was reported, providing further evidence for safety.^8^

TIPS trial eligibility criteria state that arterial occlusion, whether partial or complete, must be visualized for inclusion in the treatment arm while adult stroke guidelines do not require visualization of arterial occlusion on imaging prior to administering IV tPA.^7^ This criterion stems from safety priorities of the TIPS trial given higher rates of stroke mimics in children.^7^ In our case, diffusion-weighted imaging changes confirmed acute, focal areas of ischemia consistent with clinical findings. As large vessel occlusion was excluded by MRA, persistent occlusion of perforating arteries was postulated to explain the patient’s clinical presentation. We chose to extrapolate from adult literature rather than strictly adhere to the TIPS trial eligibility criteria and treated this patient with IV tPA (Figure 1). This decision was made given the severity of the patient’s deficits and confidence that the cause of these symptoms was AIS.

In the adult population, regardless of large vessel or small vessel occlusion, thrombolysis is indicated and visualization of arterial occlusion on neuroimaging is not required pretreatment, as has previously been the practice in pediatric stroke.^9^ This is further supported by a successfully used pediatric stroke protocol where large vessel occlusion was not inclusion criteria for IV tPA.^8^

In summary, our case illustrates a positive outcome following off-label use of thrombolysis for the treatment of presumed small vessel ischemic stroke in a pediatric patient. Our results do not affect current guidelines that favor thrombolysis in pediatric AIS with confirmed large vessel occlusion; however, our case supports individualized case-by-case decision making in children with AIS.

## Disclosures

Ilana Hanes, Jorge Davila, Adam Kirton, and Erick Sell report no disclosures. Serena Orr receives royalties from Cambridge University Press.
